# Publishing Without Barriers: A Vision for What Comes Next

**DOI:** 10.21627/6ejz6q37

**Published:** 2026-01-31

**Authors:** Kara L. Lynch, Timothy J. Garrett, Christopher D. Herold

**Affiliations:** Department of Laboratory Medicine, UCSF ZSFG Clinical Laboratory, 1001 Potrero Avenue, NH2M, San Francisco, CA 94110, USA; Department of Pathology, Immunology and Laboratory Medicine University of Florida, College of Medicine, P.O. Box 100275, Gainesville, FL 32610-0275, USA; MSACL, 205 12th St., Del Mar, CA 92014

## Abstract

JMSACL has partnered with Stanford University Press and moved to a non-profit, open access model to better support trustworthy, widely available research in laboratory medicine. Publishing and reading will remain free in 2026, with institutions helping fund open access from 2027. MSACL will retain ownership of the journal, meaning all existing relationships (e.g., PubMed Central indexing) and scoring histories (e.g., Impact Factor) will remain intact. The use of AI in collaboration with human oversight to expedite review speed, while maintaining the quality of published content, will be evaluated for incorporation into the publishing workflow.

The *Journal of Mass Spectrometry and Advances in the Clinical Lab (JMSACL)* enters a new chapter with this issue. We are pleased to announce a publishing partnership with Stanford University Press (SUP), which, in collaboration with the Public Knowledge Project (PKP), is focused on expanding sustainable Open Access (OA) models of non-profit journal publishing [1]. This transition represents something larger than a pedestrian change in publisher. We are moving from a commercial publishing environment to one rooted in academic values and a non-profit mission that reflects our commitment to accessibility, scientific integrity, and meaningful impact in laboratory medicine.

Because Mass Spectrometry and Advances in the Clinical Lab (MSACL) retains ownership of the journal, all existing relationships—including our Impact Factor, CiteScore, and indexing connections—remain fully intact. What changes is the framework in which we operate. For 2026, *JMSACL* will be supported by grant funding secured by the SUP+PKP partnership from The Gates Foundation and operate under a Diamond Open Access model with no article processing charges (APCs) for authors or subscription fees for readers. Beginning in 2027, *JMSACL* is planning to transition to the Subscribe to Open (S2O) model, in which institutional subscriptions collectively support the costs of publication, allowing all articles to remain openly available to all readers, without APCs. SUP has also announced a partnership with Knowledge Unlatched to coordinate and manage library subscription services for its S2O journals, further strengthening the non-profit infrastructure supporting this model [2]. This approach aligns community support with barrier-free dissemination, reinforcing the shared responsibility of the research community in sustaining high-quality scholarly publishing.

## Publishing Beyond Metrics

Traditional metrics such as Impact Factor, though imperfect, remain relevant markers of visibility and reach. Yet the true value of *JMSACL* is whether the work published within our pages advances laboratory practice, improves patient outcomes, accelerates the translation of new technologies into clinical care, and supports the next generation of clinical laboratory scientists. Our partnership with SUP+PKP, combined with our commitment to OA, will allow us to focus on these deeper measures of impact while maintaining the discoverability and recognition authors need for professional advancement.

## Innovation in Editorial Practice

As part of this transition, *JMSACL* is adopting carefully governed, artificial intelligence (AI)-assisted workflows under close human supervision. Large language models will support defined technical tasks to help streamline administrative processes and reduce turnaround times. We will also explore the use of customized, AI-prepared review summaries that may be accessed by peer reviewers as a supplemental resource under the strict guidance that any AI-generated content should not be referenced or used without human-based expert validation.

These novel materials are intended to support, not replace, expert judgment. They do not constitute peer review on their own. All manuscripts will continue to undergo full expert evaluation and editorial oversight, with reviewers retaining complete discretion over how—or whether—such materials inform their assessment. Importantly, all AI tools deployed by *JMSACL* operate under business agreements that explicitly prohibit the use of submitted content for model training. Manuscript confidentiality and data privacy remain paramount. Furthermore, AI tools will not be used by editorial staff for either copyediting or composition. We anticipate that this hybrid approach—combining advanced tools with expert human oversight—will enhance efficiency and reviewer support without compromising rigor, integrity, or trust.

## Looking Ahead

Laboratory medicine stands at an inflection point. Advances in mass spectrometry, multi-omics integration, AI, and precision diagnostics are reshaping clinical care in ways that would have seemed improbable a decade ago. The laboratory is no longer a support function operating at the margins of clinical practice; it is central to clinical decision-making and the future of personalized medicine.

*JMSACL* exists to document, accelerate, and shape this transformation. Our partnership with SUP+PKP, our transition to sustainable OA, and our commitment to responsible editorial innovation position the journal to serve the global laboratory medicine community more effectively than ever before. We invite you to join us by submitting your most impactful work, by serving as a reviewer, by engaging with published research, and by urging your institutions to subscribe to support. Together, we will build a publication that both reflects the state of laboratory medicine science and actively advances it.
